# Optimization of Innovative Three-Dimensionally-Structured Hybrid Vesicles to Improve the Cutaneous Delivery of Clotrimazole for the Treatment of Topical Candidiasis

**DOI:** 10.3390/pharmaceutics11060263

**Published:** 2019-06-06

**Authors:** Maria Letizia Manca, Iris Usach, José Esteban Peris, Antonella Ibba, Germano Orrù, Donatella Valenti, Elvira Escribano-Ferrer, Juan Carmelo Gomez-Fernandez, Francisco José Aranda, Anna Maria Fadda, Maria Manconi

**Affiliations:** 1 Department Scienze della Vita e dell’Ambiente, University of Cagliari, Via Ospedale 72, 09124 Cagliari, Italy; valenti@unica.it (D.V.); mfadda@unica.it (A.M.F.); manconi@unica.it (M.M.); 2 Department of Pharmacy and Pharmaceutical Technology and Parasitology, University of Valencia, Burjassot, 46100 Valencia, Spain; Iris.Usach@uv.es (I.U.); Jose.E.Peris@uv.es (J.E.P.); 3 Department of Surgical Science, University of Cagliari, Molecular Biology Service Lab (MBS), Via Ospedale 40, 09124 Cagliari, Italy; ibba@unica.it (A.I.); germanoorru@gmail.com (G.O.); 4 Biopharmaceutics and Pharmacokinetics Unit, Institute for Nanoscience and Nanotechnology, University of Barcelona, 08028 Barcelona, Spain; eescribano@ub.edu; 5 Department of Biochemistry and Molecular Biology A, Regional Campus of International Excellence Campus Mare Nostrum, University of Murcia, 30080 Murcia, Spain; jcgomez@um.es (J.C.G.-F.); fjgomez@um.es (F.J.A.)

**Keywords:** phospholipid vesicles, clotrimazole, co-solvents, skin delivery, fungal infections, *Candida albicans*

## Abstract

New three-dimensionally-structured hybrid phospholipid vesicles, able to load clotrimazole in a high amount (10 mg/mL), were obtained for the first time in this work by significantly reducing the amount of water (≤10%), which was replaced with a mixture of glycerol and ethanol (≈90%). A pre-formulation study was carried out to evaluate the effect of both the composition of the hydrating medium and the concentration of the phospholipid on the physico-chemical properties of hybrid vesicles. Four different three-dimensionally-structured hybrid vesicles were selected as ideal systems for the topical application of clotrimazole. An extensive physico-chemical characterization performed using transmission electron microscopy (TEM), cryogenic transmission electron microscopy (cryo-TEM), ^31^P-NMR, and small-angle X-ray scattering (SAXS) displayed the formation of small, multi-, and unilamellar vesicles very close to each other, and was capable of forming a three-dimensional network, which stabilized the dispersion. Additionally, the dilution of the dispersion with water reduced the interactions between vesicles, leading to the formation of single unilamellar vesicles. The evaluation of the in vitro percutaneous delivery of clotrimazole showed an improved drug deposition in the skin strata provided by the three-dimensionally-structured vesicles with respect to the commercial cream (Canesten^®^) used as a reference. Hybrid vesicles were highly biocompatible and showed a significant antifungal activity in vitro, greater than the commercial cream Canesten^®^. The antimycotic efficacy of formulations was confirmed by the reduced proliferation of the yeast cells at the site of infection in vivo. In light of these results, clotrimazole-loaded, three-dimensionally-structured hybrid vesicles appear to be one of the most innovative and promising formulations for the treatment of candidiasis infections.

## 1. Introduction

The skin is the main barrier and defence of our body and, for this reason, it is susceptible to different infections and inflammations. Fungal infections, especially candidiasis, currently represent a significant problem for human health worldwide [1]. Moreover, *Candida albicans* (*C. albicans*) is able to penetrate the *stratum corneum* inducing lesions, which often becomes systemic and occasionally lethal [2]. Human cutaneous candidiasis is a pustular dermatosis characterized by visible and microscopic abscesses that are predominantly composed of polymorphonuclear leukocytes. Usually, the effectiveness of a topical antifungal treatment depends on the ability of the drug to persist in the skin surface, penetrate through the *stratum corneum*, and through the *C. albicans* biofilm’s matrix to achieve the effective drug concentration levels inside the yeast’s cells [3]. Different types of topical compounds have been used in the treatment of fungal skin infections. Among the main classes of antifungal drug, the azoles, which inhibit the fungal cytochrome P450, are the most successful in clinical use since the late 1960s [4]. Clotrimazole (1-[(2-chlorophenyl)diphenylmethyl]-1H-imidazole), an imidazole derivative, is a widely used drug with a wide spectrum of antifungal activity, particularly against candidiasis. Clotrimazole is orally administered for the treatment of systemic candidiasis (pulmonary and disseminated cryptococcosis, and aspergillosis). However, because of its adverse effects following systemic administration, it is mainly used for the treatment of localized topical candidiasis. Further, its topical effectiveness seems to be strongly affected by the formulation [5,6], which may play a key role in ensuring the permanence and promoting the penetration of drugs in the skin and biofilm’s matrix [7,8]. To reach this goal, during the past few decades, new topical delivery systems have been explored [9,10,11]. Amongst them, phospholipid vesicles have evoked a considerable interest. Their effectiveness in skin delivery was reported for the first time by Mezei and Gulasekharam in 1980 [12]. In the last two decades, intensive research led to the introduction of new classes of liposome-like vesicles, capable of improving the performances of conventional liposomes, such as elastic or ultra-deformable liposomes so called Transfersomes^®^ [13,14,15]; soft vesicles containing ethanol (ethosomes) [16,17,18,19]; high performant vesicles containing penetration enhancers (penetration enhancers containing vesicles, PEVs) [20]; vesicles containing high amount of glycerol in the water phase (glycerosomes) [21,22,23,24]; and vesicles immobilized in a network of hyaluronan sodium salt (hyalurosomes) [25,26,27]. All these phospholipid vesicles are characterized by the presence of at least one additive component or a water cosolvent in the formulation and are mainly composed of phospholipids and not less than 50% of water as a hydrating medium.

To the best of our knowledge, in the present work, for the first time, new phospholipid vesicles were developed by significantly reducing the amount of water (≤10%) in the mixture used as a hydrating medium. Instead of water, a mixture of two co-solvents, glycerol and ethanol, was used. The assembling of phospholipid in a medium with a reduced amount of water was never studied before, and seems to be peculiar as the obtained dispersion appeared yellow-brown-transparent and highly viscous [28]. Clotrimazole was stably loaded in high amounts into these hybrid vesicles and their main physico-chemical characteristics (size, size distribution, and surface charge) were measured. Morphology and structure were evaluated by using transmission electron microscopy (TEM), cryogenic transmission electron microscopy (cryo-TEM), ^31^P nuclear magnetic resonance (^31^P-NMR), and small-angle X-ray scattering (SAXS). The topical delivery of clotrimazole containing hybrid vesicles was studied in vitro using Franz diffusion cells and newborn pig skin. The vesicle biocompatibility was assessed in vitro using keratinocytes. Further, the effectiveness of clotrimazole-loaded vesicles against different *Candida* strains was studied in vitro and in vivo against *C. albicans* infections on mice.

## 2. Materials and Methods

### 2.1. Materials

Lecithin and glycerol were purchased from Galeno (Potenza, Italy). Clotrimazole, ethanol, and all the other products of analytical grade were purchased from Sigma-Aldrich (Milan, Italy). Cell medium, fetal bovine serum, penicillin, streptomycin, and all the other reagents for cell studies were purchased from Life Technologies Europe (Monza, Italy).

### 2.2. Vesicle Preparation

Vesicles were prepared using a two-step method avoiding the use of toxic organic solvents. Lecithin (900 mg) and clotrimazole (100 mg) were left to swell overnight using different hydrating mixtures (5 mL): glycerol/ethanol/water 59:39:2 (59:2 vesicles); glycerol/ethanol/water 53:37:10 (54:10 vesicles); glycerol/ethanol/water 69:29:2 (69:2 vesicles) and glycerol/ethanol/water 63:27:10 (63:10 vesicles). After that time, each dispersion was sonicated (15 cycles 5 sec on and 2 sec off, 13 microns of probe amplitude) with a high intensity ultrasonic disintegrator (Soniprep 150, MSE Crowley, London, U.K.). Then, a further aliquot (5 mL) of the appropriate hydrating mixture was added to each dispersion, which was then sonicated for another 15 cycles (5 sec on and 5 sec off, 13 microns of probe amplitude) to obtain the three-dimensionally-structured hybrid vesicles. The composition of samples is reported in Table 1. Empty vesicles were also prepared to evaluate the effect of the drug on vesicle assembling.

Samples were purified from the non-incorporated clotrimazole by dialysis using dialysis tubing (Spectra/Por^®^ membranes: 12–14 kDa MW cut-off, 3 nm pore size; Spectrum Laboratories Inc., DG Breda, The Netherlands). Each sample (1 mL) was dialyzed for 4 h, under continuous stirring, against the appropriate glycerol/ethanol/water mixture (2.5 l). The system was maintained at 25 °C and the medium was refreshed every hour to ensure the complete removal of the unentrapped drug.

### 2.3. Vesicle Characterization

Transmission electron microscopy (TEM) and cryogenic transmission electron microscopy (cryo-TEM) analyses were used to confirm the vesicle formation and evaluate the morphology. TEM analyses were performed using a JEM-1010 (Jeol Europe, Paris, France) transmission electron microscope equipped with a digital camera MegaView III and a Software “AnalySIS,” at an accelerating voltage of 80 kV. Before the analyses, samples were stained with phosphotungstic acid solution (1%). 

Cryo-TEM analyses were performed to evaluate the morphology of vesicles after dilution with water. A total of 5 μL of the vesicle dispersion were placed on a glow-discharged holey carbon grid, vitrified using a Vitrobot (FEI Company, Eindhoven, The Netherlands), and analyzed using a Tecnai F20 TEM (FEI Company) microscope. The sample was observed in a low-dose mode, at 200 kV, and at a temperature of around −173 °C.

A photon correlation spectroscopy method was used to evaluate the average diameter and polydispersity index (a measure of the size distribution width) by using a Zetasizer nano-ZS (Malvern Instruments, Worcestershire, U.K.). Samples were backscattered using a helium-neon laser (633 nm) at an angle of 173° and a constant temperature of 25 °C. The Zetasizer nano-ZS was also used to measure the zeta potential by means of the M3-PALS (mixed mode measurement-phase analysis light scattering) technique, which measures the particle electrophoretic mobility [29]. Just before the size distribution and zeta potential analyses, the samples (100 μL) were diluted with the hydrating mixture used for the preparation (10 mL). 

The vesicle average size and zeta potential were monitored over 90 days of storage at room temperature (25 ± 1 °C) to evaluate the stability of the samples.

The entrapment efficiency (EE) was calculated as the percentage of the amount of clotrimazole after dialysis versus that initially used. The clotrimazole concentration was measured using high performance liquid chromatography (HPLC) after disruption of the vesicles with methanol (1/1000 dilution). A chromatograph Alliance 2690 (Waters, Milano, Italy) equipped with a photodiode array detector and a computer integrating apparatus (Empower^TM^ 3) was used to measure the amount of clotrimazole. A Sunfire C18 column (5 μm, 4.6 × 150 mm, Waters) was used for the analysis, which was performed at 211 nm. A mixture of water, methanol, and acetonitrile (30/5/65 *v*/*v*), delivered at a flow rate of 1 mL/min, was used as the mobile phase.

### 2.4. ^31^P-NMR Measurements 

A Bruker Avance 600 instrument (Bruker, Etlingen, Germany) was used to process the samples operating at 242.9 MHz to collect ^31^P-NMR spectra. The spectra were obtained in the presence of a gated broad band proton decoupling (5 W input power during acquisition time), and accumulated free inductive decays were obtained from up to 8000 scans. A spectral width of 48,536 Hz, a memory of 48,536 data points, a 2 s interpulse time, and a 90° radio frequency pulse (11 μs) were used with inverse-gated decoupling ^1^H. Prior to the Fourier transformation, an exponential multiplication was applied, resulting in a 100 Hz line broadening.

### 2.5. Small- and Wide-Angle X-ray Scattering 

A modified Kratky compact camera (Hecus MBraun-Graz-Optical Systems, Graz, Austria), was used to simultaneously measure the small- (SAXS) and wide- (WAXS) angle X-ray scattering. The instrument used two coupled linear position sensitive detectors (PSD, MBraun, Garching, Germany) to monitor the s-ranges (*s* = 2sin*θ*/*λ*, 2*θ* = scattering angle, *λ* = 1.54 Å). Nickel-filtered Cu K*α* X-rays were generated using a Philips PW3830 X-ray Generator operating at 50 kV and 30 mA. Ag-stearate (small-angle region, *d*-spacing at 48.8 Å) and lupolen (wide-angle region, *d*-spacing at 4.12 Å) were used as reference materials for the calibration of the position of the detectors. The samples were loaded in a thin-walled, high-quality quartz capillary (1 mm diameter) held in a steel cuvette, which provided good thermal contact to the Peltier heating unit. Data analysis was performed with 3D-View v4.1 software (Hecus MBraun, Graz, Austria).

The program GAP (Global Analysis Program) [30,31] was used to analyze the background of corrected SAXS data, which allowed for retrieving the membrane thickness [d_B_ = 2(z_h_ + 2*σ*_h_)] from a full *q*-range analysis of the SAXS patterns. The parameters z_h_ and σ_h_ (position and width of the Gaussian) were used to describe the electron-dense head group regions within the electron density model.

### 2.6. In Vitro Skin Delivery Studies

Experiments were performed using new-born pig skin sandwiched between the donor and receptor compartments of Franz vertical cells with an effective diffusion area of 0.785 cm^2^. The receptor compartment was filled with saline (NaCl 0.9% in water), thermostated at 37 ± 1 °C, and continuously stirred. Clotrimazole containing three-dimensionaly-structured hybrid vesicles was applied (100 μL) onto the skin surface under non-occlusive conditions [32]. Canesten^®^ cream was used as a reference. The receiving solution was withdrawn at regular intervals (2, 4, 6, and 8 h), replaced with pre-thermostated (37 ± 1 °C) fresh saline solution, and analyzed using HPLC for clotrimazole content (Section 2.3). At the end of the experiment, the skin surface was gently blotted on filter paper, and adhesive tape Tesa^®^ AG (Hamburg, Germany) was used to remove the *stratum corneum* via stripping. The epidermis was separated from the dermis with a surgical scalpel. Tape strips, epidermis, and dermis were cut into small pieces to increase the surface contact with methanol and were sonicated for 2 min in an ice bath to completely extract the drug. The tapes and tissues were filtered out and the solutions were assayed for drug content using HPLC (see Section 2.3).

### 2.7. Cell Viability Assay

Human keratinocytes (HaCaT) (ATCC collection, LCG Standards S.R.L, Sesto San Giovanni, MI, Italy) were grown as monolayers in 75 cm^2^ flasks incubated in 100% humidity and 5% CO_2_ at 37 °C. Dulbecco’s Modified Eagle Medium (DMEM) with high glucose, enriched with 10% fetal bovine serum, penicillin-streptomycin, and fungizone, was used as the growing medium. For the experiments, cells were seeded in a 96-well plates at a density of 7.5 × 10^3^ cells/well, and after 24 h of incubation, were exposed for 48 h to clotrimazole-loaded hybrid vesicles at different drug concentrations (2, 10, 20, 50, 100 μg/mL). Cell viability was assessed by adding (100 µL, 0.5 mg/mL) of MTT [3(4,5-dimethylthiazolyl-2)-2, 5-diphenyltetrazolium bromide] to each well. After 3 h, dimethyl sulfoxide (100 μL in each well) was used to dissolve the formazan crystals, and the absorbance was read at 570 nm using a microplate reader (Multiskan EX, Thermo Fisher Scientific, Inc., Waltham, MA, USA). All the experiments were repeated at least three times, each in triplicate. The results are presented as the percentage of untreated cells (100% viability) [25,33,34].

### 2.8. In Vitro Antifungal Activity

A clinical *Candida albicans* multidrug-resistant isolate CA97, was used for the antifungal susceptibility test. This strain has been previously characterized for its response to different commercial antifungals and it was shown to be resistant to three different azoles (Fluconazole, Voriconazole, Ketoconazole) [35]. This clinical isolate has been subsequently evaluated in vitro following the methodology recommended by the Clinical and Laboratory Standards Institute (CLSI) [36], and it was shown to be clotrimazole sensitive (data not shown in this manuscript). 

The agar diffusion method was performed by using the Kirby–Bauer procedure, used as a preliminary antimicrobial test to reveal the antifungal susceptibility profile in the formulations [37,38]. Colony forming cells (CFU, 1 × 10^7^/mL) were inoculated onto the surface of a Sabouraud agar plate and 50 µL of each formulation, corresponding to 500 μg of clotrimazole, was inserted in each well (Ø10 mm diameter and 2 mm thick) in the centre of the plate [39]. Petri dishes were then incubated in air at 37 °C for 24 h. After incubation, the inhibition diameter was measured. The experiment was performed in triplicate.

The minimum inhibitory concentration (MIC) and minimum bactericide concentration (MBC) were measured according to the methodologies recommended by the Clinical Laboratory and Standards Institute [36]. The experiment has been performed in sterile Nunc^TM^ Microwell^TM^ 96-well microplates (Thermo Fisher Scientific). Each formulation (200 L) serially diluted (1:2) in Sabouraud dextrose broth (Microbiol Uta, Cagliari, Italy) from 5 to 2.4 × 10^−3^ µg/mL of clotrimazole was tested. Suspension of *C. albicans* (200 μL, 1 × 10^6^ CFU/mL) diluted in Sabouraud dextrose broth was inoculated into each well to obtain a final inoculum concentration of 10^5^ CFU/mL. After 48 h at 37 °C, the plates were read with a microplate reader at 620 nm (SLT-Spectra II, SLT Instruments, Crailsheim, Germany). The MIC was the lowest concentration of an antimicrobial that inhibited the visible growth (absence of turbidity). 

To determine the MBC, 100 μL of the dilution representing the MIC and at least two of the more concentrated tested clotrimazole formulations were plated in Sabouraud agar at 37 °C; after 24 h, the colony-forming units (CFUs) were enumerated. The MBC was the lowest concentration able to effectively reduce the yeast growth (99.5%). 

The ability of clotrimazole-containing, three-dimensionally-structured hybrid vesicles to inhibit the biofilm formation was evaluated following the crystal-violet staining protocol, as previously reported [39,40]. Inoculum and the formulation dilution procedures were the same as those already described for the MIC and MBC experiments. In this context, the minimum biofilm inhibitory concentration (MBIC) represented the lowest concentration able to interfere with biofilm formation. In other words, after the *C. albicans* biofilm staining using crystal violet, the MBIC represented the concentration of drug able to show an absorbance comparable to the negative control (sample without bacteria). For each formulation, the experiment was performed in triplicate. For the same concentration, all data that showed a standard deviation within ±10% of the mean value were considered significant.

### 2.9. In Vivo Antifungal Activity

Protocols for the in vivo studies using mice were approved by the Animal Care Committee of the Faculty of Pharmacy at the University of Valencia (Spain) (reference: 2016/VSC/PEA/00208). Male 2–3 months old ICR (CD-1) mice, weighing 20–25 g, (Envigo, the provider of standard research models, Barcelona, Spain), were obtained from the animal facility of the University of Valencia (Faculty of Pharmacy) and were kept in a clean room at a temperature of 23 ± 1 °C, a relative humidity of 60%, and a light/dark cycle of 12 h. A standard laboratory diet was administered to the mice, which had access to water ad libitum. Prior to fungal infection, mice were immunosuppressed by means of intraperitoneal injections of cyclophosphamide (100 mg/kg/day) for 3 days [41,42]. 

A working culture of *C. albicans* grown for 48 h at 35 °C on Sabouraud dextrose agar was used to prepare a yeast suspension of colony forming units (CFU 10^7^/mL) in a mixture (50/50) of cell culture medium RPMI 1640 and a yeast extract-peptone-dextrose medium. 

Each animal’s back was shaved with an electric clipper on the second day of treatment with cyclophosphamide. On day 3, the *C. albicans* (100 μL) suspension was applied on the shaved skin using a specially designed cylindrical plastic device (4.5 mm i.d. × 6 mm) stuck in the animal’s back with a cyanoacrylate adhesive to keep the suspension in contact with the skin as long as possible but allowing aerobic conditions. After 24 h of the inoculation of *C. albicans*, the infected area was treated with the different formulations (50 μL): saline (control), Canesten^®^ (reference), and clotrimazole-loaded 59:2 and 63:10 vesicles. All animals were sacrificed after 24 h and the skin around the application area was excised. The surface of the excised skin was scraped, suspended with 1 mL of Eugon LT 100 broth in an Eppendorf tube, vortexed for 30 s, and centrifuged (2000× *g*, 5 min) to collect the pellet containing the *C. albicans*. The supernatant was disposed and the pellet was resuspended with an additional volume of Eugon LT 100 broth (1 mL), vortexed, and centrifuged as indicated. The pellet obtained after the two washes was resuspended in 1 mL of Eugon LT 100 broth and decimal dilutions were prepared with the same broth. Each dilution (100 μL) was mixed with 15–20 mL of molten Sabouraud dextrose chloramphenicol agar in a Petri dish (two dishes for each dilution) and incubated at 35 °C for 48–72 h. The total CFU count of *C. albicans* on the skin samples was calculated from the number of CFU in dishes with 30–300 CFU, considering the dilution factor and the volume of dilution in the dishes.

### 2.10. Microscopic Visualization of Skin Infected with C. albicans

Skin samples of mice inoculated with *C. albicans* were examined after 24 h using a scanning electron microscope (SEM, S-4800 Hitachi, Madrid, Spain), and compared with skin samples of untreated mice to evaluate the effectiveness of the infection. Samples were fixed using a paraformaldehyde and glutaraldehyde solution (Karnovsky’s Fixative) and dehydrated in a series of ethanol washes (70, 90, 95, and 100%, *v*/*v*). After drying using a critical point method, samples were coated with gold-palladium prior to microscopic visualization.

### 2.11. Statistical Data Analysis 

Results are expressed as mean ± standard deviation. Statistical differences between groups were evaluated by using multiple comparison of means (ANOVA), while results between groups were analyzed by using the Student–Newmans–Keuls method. Significance was tested at the 0.05 level of probability (*p*) and data analysis was carried out using the IBM SPSS Statistics v.24 (IBM Corp., Armonk, NY, USA) software.

## 3. Results

### 3.1. Characterization of Three-Dimensionally-Structured Hybrid Vesicles

Aiming at improving the loading and stability of clotrimazole in phospholipid vesicles, a pre-formulation study was performed to find an adequate and promising preparation. Phospholipid vesicles were prepared using different kinds and amounts of phospholipids, and adding surfactants, co-solvents, or other additives. The obtained vesicles were unable to retain clotrimazole for more than one day as it precipitated in a short time irrespective of the composition and the size of the vesicles. For this reason, for the first time in this work, innovative formulations were developed, which involved reducing the amount of water usually used as typical hydrating medium of phospholipid vesicles, and mainly replacing it (≈90%) with a mixture of ethanol and glycerol, thus obtaining yellow-brown-transparent and viscous dispersions, which avoided the drug precipitation problem for a long time. Different ratios of glycerol, ethanol, and water were tested, and four mixtures were selected as they seemed to be the most promising in terms of size distribution and clotrimazole retention (Table 1). Corresponding empty vesicles were prepared to evaluate the influence of clotrimazole in vesicle assembling. 

**Figure 1:**
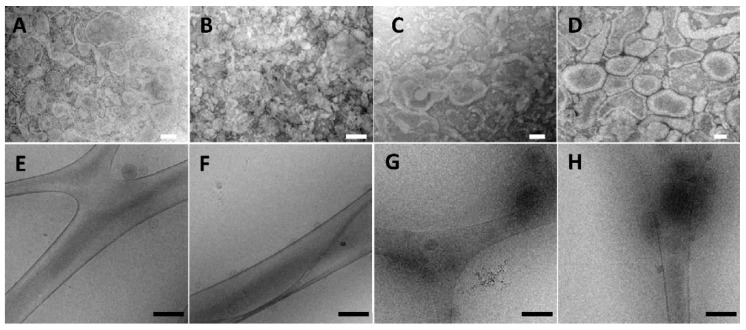
TEM images (upper panel) of three-dimensionally-structured hybrid 59:2 vesicles (**A**), 53:10 vesicles (**B**), 69:2 vesicles(**C**), and 63:10 vesicles (**D**). Scale bars represent 100 nm. Cryo-TEM images (lower panel) of diluted three-dimensionally-structured hybrid 59:2 vesicles (**E**), 53:10 vesicles (**F**), 69:2 vesicles (**G**), and 63:10 vesicles (**H**). Scale bars represent 500 nm.

Due to the novelty of these formulations, it is important that they were fully characterized. First, the formation and morphology of vesicles in their native environmental was observed using TEM (Figure 1, upper panel). The images confirmed the presence of coexisting multi- and single-lamellar vesicles, characterized by a fairly regular spherical shape, which appeared close-packed and formed a three-dimensional network [43]. 

Further, to simulate the behavior that the vesicles may undergo in vivo in the biological fluids, the samples were diluted (1/500) with water, and in these conditions, it was possible to freeze the dispersion and observe them by using the cryo-TEM technique. After the dilution, the three-dimensionally-structured hybrid vesicles became single vesicles, and multi- and single-lamellar structures coexisted together (Figure 1, lower panel).

The size distribution, zeta potential, and entrapment efficiency of empty and clotrimazole-loaded hybrid vesicles were measured (Table 2). Empty vesicles were bigger than the corresponding clotrimazole-loaded vesicles denoting the influence of the drug in favoring the bilayer assembling, which resulted in a larger curvature and smaller vesicles. The amount of water was also able to modify the bilayer assembling, being bigger the vesicles containing the higher amount of water (53:10 vesicles and 63:10 vesicles, *p* < 0.05) with respect to the corresponding vesicles containing the lower amount (59:2 vesicles and 69:2 vesicles). Considering the clotrimazole loaded vesicles, the mixture glycerol/ethanol/water 59:39:2, containing the lower amount of water, provided the formation of the smallest vesicles (96 ± 2 nm), while increasing the amount of water (to glycerol/ethanol/water 53:37:10) provided a significant increase in vesicle mean diameter (186 ± 6 nm). The variation of glycerol and ethanol ratio, reducing the amount of ethanol in favor of that of glycerol (69:29:2 and 63:27:10 ratio), allowed for the formation of vesicles with an intermediate size (≈135 nm), irrespective of the amount of water used. The zeta potential was highly negative for all the formulations irrespective of the presence of the drug.

The amount of clotrimazole incorporated in both formulations containing the smaller amount of water (59:2 and 69:2 vesicles) was slightly higher (≈97%) in comparison with that incorporated in the formulations containing a higher amount of water (≈83%), probably because both glycerol and ethanol favored the solubilization of the drug inside the vesicles and in the vesicle bilayer [44].

**Figure 2:**
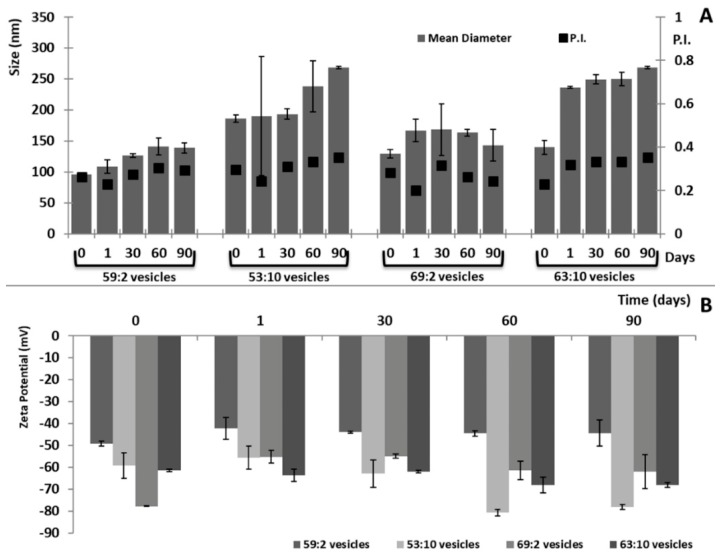
Values of size, polydispersity index (**A**), and zeta potential (**B**) of clotrimazole loaded three-dimensionally-structured hybrid vesicles collected over 90 days of storage at 25 ± 1 °C. Mean values ± standard deviation (error bars) were reported from six independent samples.

Vesicle stability was evaluated for 90 days at room temperature (25 ± 1 °C, Figure 2). The values of the checked parameters (mean diameter, polydispersity index, and zeta potential) of 59:2 and 69:2 vesicles remained almost constant over all the storage periods (variation < 10%, *p* > 0.05), while 53:10 and 63:10 vesicles, containing a higher amount of water, suffered a significant change in size, as they reached ≈238 nm in diameter in a short time. The presence of water probably reduced the stability of the vesicles favoring its aggregation or fusion.

### 3.2. ^31^P-NMR Measurements and Small- and Wide-Angle X-ray Scattering (SAXS and WAXS)

**Figure 3:**
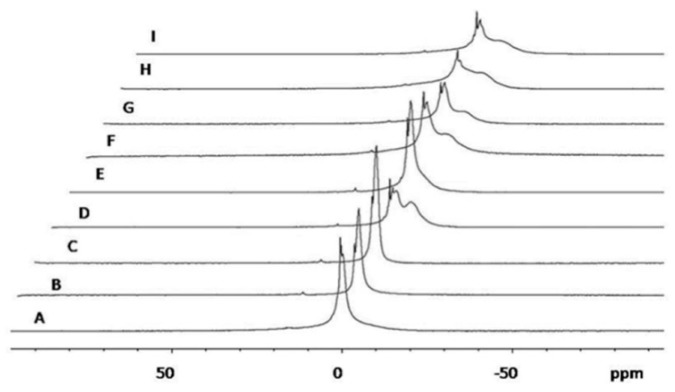
Representative spectra of empty liposomes (**A**), empty (**B**) and clotrimazole-loaded (**C**) 59:2 vesicles, empty (**D**) and clotrimazole-loaded (**E**) 53:10 vesicles, empty (**F**) and clotrimazole-loaded (**G**) 69:2 vesicles, and empty (**H**) and clotrimazole-loaded (**I**) 63:10 vesicles. Spectra are presented with a horizontal offset toward the right of 5 ppm to facilitate a clear view.

The ^31^P-NMR spectra of empty and clotrimazole-loaded three-dimensionally-structured hybrid vesicles were collected and compared (Figure 3).

The spectrum of the lecithin vesicles in water was used as a reference (Figure 3A). It showed isotropic components and a couple of narrow peaks were superimposed into a broader one, indicating the presence of vesicles of different sizes, although since the broadest one had a width at half height of about 2 ppm, it can be deduced that all these vesicles had sizes around 100 nm [45]. A similar result was obtained for empty and clotrimazole-loaded 59:2 vesicles (Figure 3B,C). These results indicate that the presence of clotrimazole did not significantly alter either size or assembling of these vesicles. However, a different pattern was observed for empty 53:10 vesicles, in which a wider peak indicative of vesicles of higher diameter, possibly >200 nm [45], was observed in addition to the isotropic peaks (Figure 3D). This may indicate that the presence of a higher amount of water may determine the appearance of bigger vesicles, possibly due to fusion of the smaller ones. Clotrimazole-loaded 53:10 vesicles (Figure 3E), shown an isotropic pattern similar to those of Figure 3A, B, or C, indicates that the presence of clotrimazole modified the size distribution of the vesicles, making them smaller than the corresponding empty ones. However, the spectrum of empty 69:2 and 63:10 vesicles (Figure 3F,H) was similar to that of Figure 3D, with the presence of vesicles ≈200 nm together with isotropic ones, indicating that a higher percentage of glycerol and a lower amount of ethanol modified the bilayer assembling and vesicle diameter, in the same manner that the water allowed the formation of bigger vesicles. Similar spectra were observed for clotrimazole-loaded 69:2 and 63:10 vesicles (Figure 3G,I), which also indicate in this case, the presence of a heterogeneous population of vesicles. However, it should be stated that when ^31^P-NMR is used to calculate the size of lipid vesicles, it usually gives a lower diameter than those given using electron microscopy, which can be explained, at least in part, by ellipsoidal deformation during NMR measurements [45].

To further characterize the vesicle structure, small- and wide-angle X-ray scattering (SAXS and WAXS) were performed. Empty liposomes, and empty and clotrimazole-loaded three-dimensionally-structured hybrid vesicles, were analyzed and compared. WAXS diffractograms of all samples showed a very broad peak, thus indicating that all vesicles were in a fluid condition (data not shown). On the other hand, the SAXS diffraction pattern of all empty and clotrimazole-containing three-dimensionally-structured hybrid vesicles displayed a broad peak with pure diffuse scattering, indicative of unilamellar vesicles where the bilayers had no fixed geometrical relationship to each other (data not shown). The GAP model allowed us to calculate the fit dimensions of the polar head group and the thickness of the bilayer (Table 3). The mean distance of the polar head to the center of the bilayer (z_H_) was similar in all the samples (changes were always not higher than 2 Å). With respect to empty liposomes, a small decrease of 1.8 Å was observed for empty 59:2 vesicles (from 18.7 to 16.9 Å), and a 1.7 Å was detected for empty 53:10 vesicles (from 18.7 to 20.4 Å). In the case of the polar head amplitude (σ_H_), the value of σ_H_ of pure phospholipid bilayer is usually around 3 Å and in agreement with that, a value of 3.4 ± 0.1 Å, was observed for empty liposomes. However, considerably higher values were found for empty (6.5 ± 0.1 Å) and clotrimazole-loaded 59:2 vesicles (6.3 ± 0.2 Å), and empty (6.7 ± 0.1 Å) and clotrimazole-loaded 63:10 vesicles (6.8 ± 0.2 Å), probably due to the high amount of glycerol and ethanol in the mixture. A substantially lower increase in σ_H_ was observed for empty (4.7 ± 0.2 Å) and clotrimazole-loaded 69:2 vesicles (4.5 ± 0.2 Å), and empty (4.8 ± 0.2 Å) and clotrimazole-loaded 63:10 vesicles (4.1 ± 0.2 Å). This different behavior may indicate that ethanol is the main substance responsible for the expansion of the polar group since 59:2 and 53:10 vesicles contained 39% and 36% of ethanol respectively, while 69:2 and 63:10 vesicles contained 29% and 27% of ethanol respectively. Results also indicate that glycerol simulated a behavior like that of water.

The bilayer thickness (d_B_) of empty liposomes was 51.0 ± 0.6 Å and those of three-dimensionally-structured hybrid vesicles were significantly higher (Table 3). This expansion of the bilayer thickness was a consequence of the presence of the glycerol and ethanol mixture, which played an important role on vesicle assembly. Indeed, the d_B_ of clotrimazole-loaded 59:2 vesicles was 60.6 ± 1.2 Å and that of clotrimazole-loaded 53:10 vesicles was 62.0 ± 1.2 Å. Additionally, ethanol seemed to be the main substance responsible of this bilayer enlargement, and as already found for the increase of σ_H_, for empty and clotrimazole-loaded 69:2 and 63:10 vesicles, the d_B_ values were slightly smaller than that of 59:2 and 53:10 vesicles, which contained a higher amount of ethanol.

### 3.3. In Vitro Skin Permeation Studies

**Figure 4:**
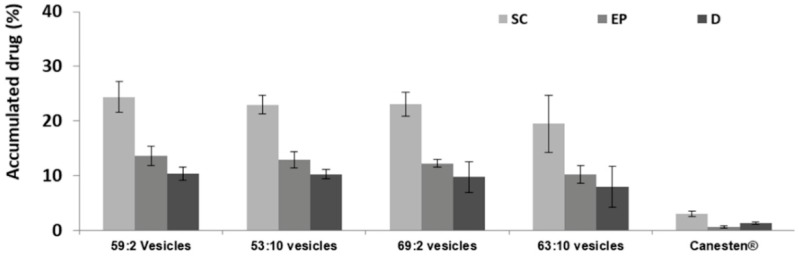
Cumulative amount of clotrimazole accumulated in the *stratum corneum* (SC), epidermis (EP), and dermis (D) after 8 h of treatment with the clotrimazole-loaded three-dimensionally-structured hybrid vesicles or Canesten^®^ cream. Bars represent the mean ± standard deviation (error bars) of at least six independent experimental determinations.

In vitro skin permeation studies displayed the ability of the three-dimensionally-structured hybrid vesicles to facilitate the accumulation of clotrimazole in the epidermis, especially in the *stratum corneum* (Figure 4). The amount of clotrimazole permeated through the skin into the receptor compartment was very low during the 8 h of experiment as it was not detectable using HPLC.

The commercial cream Canesten^®^ was used as a reference. It is a commercial product containing clotrimazole (1%) dispersed in a cream composed of sorbitan monostearate, tween 60, cetile palmitate, cetostearyl alcohol, octyldodecanol, benzyl alcohol, and water. Canesten^®^ allowed for a very low accumulation of the drug in the different skin strata (≈2%, *p* < 0.05 versus that provided by all the other vesicles) to a significantly lesser extent with respect to three-dimensionally-structured hybrid vesicles. Indeed, when clotrimazole was loaded in hybrid vesicles, its accumulation in the *stratum corneum* (≈22%) was 7 times higher than that obtained using the commercial cream, while in the epidermis and dermis, it was 10 times higher (≈12%). No significant differences were found between the different hybrid vesicles, so we can hypothesize that all samples, thanks to the affinity of phospholipids toward the interlamellar matrix of *stratum corneum*, the moisturizing effect of glycerol and the solubilizing effect of ethanol, may reduce the skin barrier function, favoring the deposition of the drug in the different skin layers. On the contrary, the application of the commercial cream for 8 h was unable to effectively alter the assembling of the lipid matrix of the skin, thus the accumulation of clotrimazole in the different strata was significantly lower. Further, in the case of the commercial cream, the accumulation of the drug in the skin layers was limited by both the barrier effect of the *stratum corneum* and its partitioning between the *stratum corneum* and the vehicle (cream) [46]. 

### 3.4. In Vitro Cytotoxicity of Clotrimazole Loaded Hybrid Vesicles

**Figure 5:**
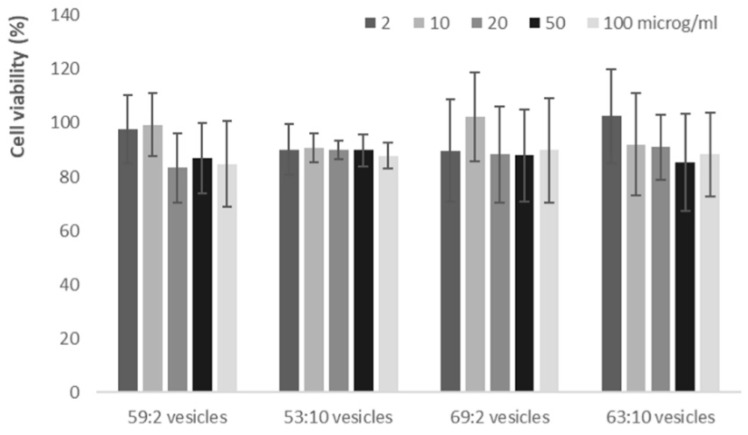
Cell viability of keratinocytes incubated for 48 h with the clotrimazole-loaded, three-dimensionally-structured hybrid vesicles. Data are reported as mean values ± standard deviation of the cell viability expressed as the percentage of the negative control (100% of viability).

The toxicity of the different clotrimazole-loaded vesicles was evaluated using keratinocytes (HaCat) and measuring the cell viability using the MTT assay (Figure 5).

Topical formulations must necessarily make contact with the keratinocytes since they are the main cells of the epidermis and it is important that their topical application do not cause any adverse effect on these cells. For this reason, in vitro biocompatibility of formulations against keratinocytes was evaluated. At all tested clotrimazole concentrations (2, 10, 20, 50, 100 μg/mL), cell viability always ranged from ≈80 to ≈100%. This indicated that the variation of the vehicle used to obtain hybrid vesicles and the significant reduction of the amount of water did not modify the high and well-known biocompatibility of phospholipid vesicles [47,48].

### 3.5. In Vitro Antifungal Susceptibility Test 

**Figure 6:**
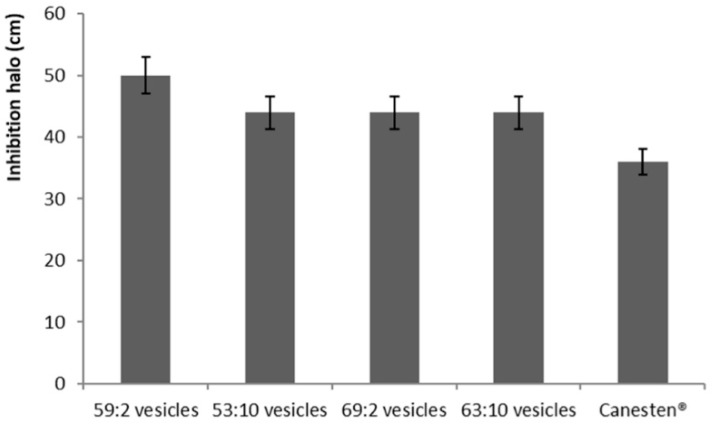
In vitro inhibition halo against *C. albicans* provided by the clotrimazole-containing, three-dimensionally-structured hybrid vesicles or Canesten^®^ cream. Data are reported as mean values ± standard deviation (error bars) of six values.

The results obtained using the Kirby–Bauer procedure displayed a significant amount of activity from clotrimazole-loaded hybrid vesicles (≈46 cm) and a lower effectiveness provided by Canesten^®^ (≈35 cm) (Figure 6), while empty vesicles did not exert any efficacy (data not shown).

The values of MIC, MBC, and antibiofilm profile were not evaluated for Canesten^®^ cream since it formed a turbid dispersion, mainly due to the presence of different insoluble functional components, which interfered with the spectrophotometric measure. The quantitative antimicrobial determination of clotrimazole-loaded hybrid vesicles indicated a different efficacy as a function of the vesicle composition. Indeed, clotrimazole-loaded 63:10 vesicles had the lowest values of MIC and MBC (1.25 μg/mL), followed by clotrimazole-loaded 59:2 vesicles (2.50 μg/mL), while 53:10 and 69:2 vesicles and all empty vesicles were characterized by higher values (>5 μg/mL) (Table 4).

An antibiofilm assay showed a different and interesting behavior because clotrimazole-loaded 59:2 vesicles provided the highest biofilm inhibition (MBIC ≤ 0.002 μg/mL), followed by clotrimazole-loaded 63:10 vesicles (MBIC = 0.004 μg/mL). These values were much lower than that provided by clotrimazole loaded 53:10 vesicles and 69:2 vesicles (MBIC = 5 μg/mL). On the contrary, empty vesicles were not able to effectively inhibit both the biofilm and growth of *C. albicans* (MBIC > 5 μg/mL). Results confirmed the effectiveness of three-dimensionally-structured hybrid vesicles to promote the ability of clotrimazole to interfere with the formation of *C. albicans* biofilm. The outcomes confirmed a great potential of clotrimazole-loaded, three-dimensionally-structured hybrid vesicles, especially 59:2 and 63:10 vesicles, in inhibiting the *C. albicans* biofilm. In addition, results indicate an important role of glycerol in promoting the effectiveness of clotrimazole in both *C. albicans* growth and biofilm formation, as the best results were obtained using 63:10 and 59:2 vesicles containing the intermediate amount of glycerol (≈0.61 mL).

### 3.6. In Vivo Antifungal Activity Evaluation

**Figure 7:**
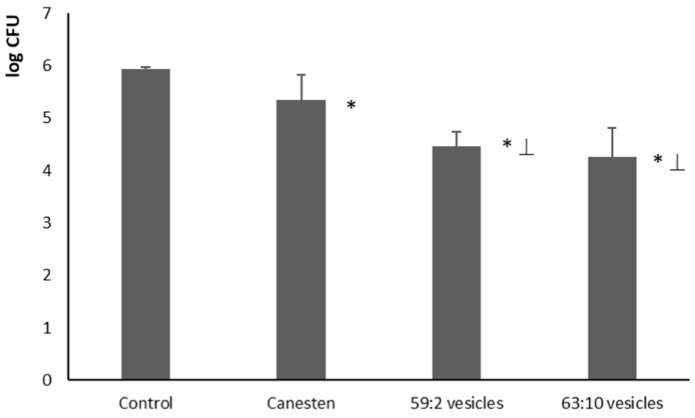
Total count of *C. albicans* (CFU) in the skin samples of mice 24 h after the application of Canesten^®^ cream and clotrimazole-loaded 59:2 and 63:10 hybrid vesicles. Values are expressed as mean ± standard deviation (*n* = 4). The symbol * indicates values that were statistically different from control group and the symbol ⊥ indicates values that were statistically different from Canesten^®^ (reference group).

The antifungal activity of clotrimazole-loaded hybrid vesicles was evaluated in vivo using mice carrying a dense layer of *C. albicans* on their dorsal skins. According to the guidelines of the ethical committee and aiming at reducing the number of used animals, only the two most promising formulations of clotrimazole-loaded, three-dimensionally-structured hybrid vesicles, which showed the best features on in vitro tests, were tested in vivo (59:2 and 63:10 vesicles). Saline was used in the control group (no inhibition of *C. albicans* growth) and Canesten^®^ cream was employed as a reference treatment. Clotrimazole vesicles, as well as Canesten^®^ cream, were able to reduce the count of the colonies of *C. albicans* (CFU) in comparison with the control group (Figure 7), but the reduction obtained using clotrimazole-loaded 59:2 and 63:10 vesicles was significantly higher (*p* < 0.05) than that obtained with Canesten^®^ cream. 

**Figure 8:**
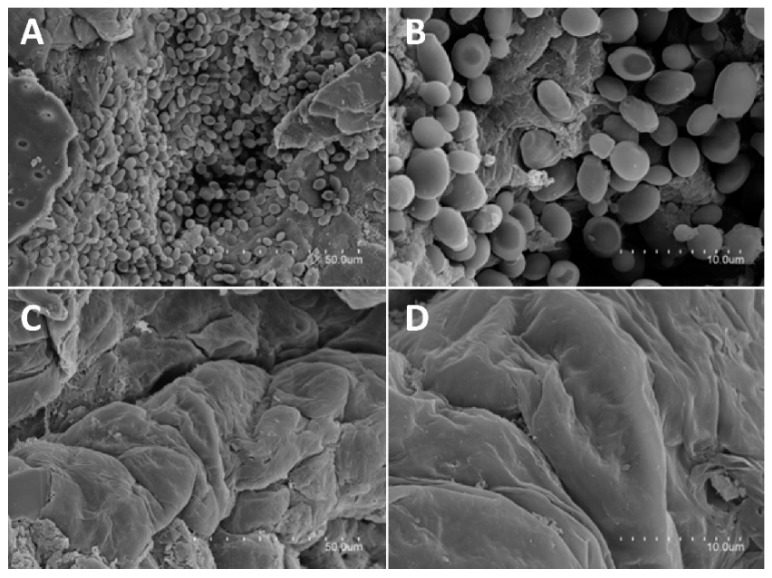
Scanning electron microscopy images of skin samples obtained from *C. albicans*-infected (**A**,**B**) and non-infected (**C**,**D**) mice. Images A and C were obtained using a 1100× magnification and B and D using a 4000× magnification.

To further investigate the effective infection of the dorsal skin of mice after treatment with *C. albicans*, SEM studies were performed (Figure 8).

Infected skin showed a large number of yeasts (10–12 microns across) adhering to the skin (Figure 8A,B), which were not visible in the untreated skin (Figure 8C,D). The images confirmed the effective proliferation of *C. albicans* and the resulting infection on the skin.

## 4. Discussion

The skin is often subjected to different infections, with fungal ones being the most frequent. They first infect the skin surface and consequently invade the *stratum corneum*. Therapeutic agents can be applied to the surface of the skin in the form of creams, lotions, or sprays. They must be able to easily penetrate the *stratum corneum* and kill the fungi (fungicidal agents), or at least reduce their ability to grow or divide (fungistatic agents). Topical treatments represent the first choice to rid the skin of topical fungi and yeasts. Azoles (i.e., Miconazole, Clotrimazole, and Ketoconazole) are especially used for the treatment of infections caused by *C. albicans*; in particular, clotrimazole topical preparations are generally well-tolerated, even if local irritation has necessitated the withdrawal of therapy in a few cases [49]. Different studies were carried out, aiming at ameliorating the antifungal efficacy of clotrimazole after topical application. Soriano et al. [50] formulated a nanoemulsion of clotrimazole designed as topical and mucosal delivery systems. Despite their good properties in terms of physico-chemical characteristics and stability behavior, these systems were not able to ameliorate the permeation parameters, nor the skin-retained amount, but were able to improve the antifungal activity in comparison with the commercial cream used as reference.

Hashem et al. [51] formulated microemulsions, which showed, when incorporated in a conventional gel, an improvement (2 or 3 times) of the clotrimazole retention in the skin, as well as of the antifungal activity, in comparison with commercial cream, probably due to the adhesive properties of the gel containing the microemulsion.

Akhtar et al. [52] demonstrated the ability of ethosomes modified using Cavamax W7 and penetration enhancers (PEG 400, isopropylmyristate, or triethanolamine), to improve the permeation and accumulation of clotrimazole into the skin. The antifungal activity against *C. Albicans* was improved as well using the ethosomal formulation. However, to ensure the correct application of the formulation in the skin, these systems were inserted in a conventional gel of Carbopol.

The insertion of a nanocarier into a structured vehicle, such as cream, ointment, or gel, to ensure the correct application of the drug in the skin, is a widely used approach. However, it must be taken into account that the diffusion and accumulation of the drug into the skin may be limited by its partitioning between the structured vehicle and the *stratum corneum*, reducing its effectiveness against topical infections.

To further ameliorate the efficacy of the treatment of the skin infections caused by *Candida albicans*, and to avoid the addition of structured vehicles, in this study, for the first time, new phospholipid vesicles were formulated by using a mixture of glycerol-ethanol with a low water content (≤10%) as a hydrating medium, leading to the formation of three-dimensionally-structured hybrid and highly viscous vesicles. Additionally, hybrid vesicles were prepared using an environmentally-friendly, easily reproducible, and organic solvent-free method. The performed dynamic laser light scattering, ^31^P-NMR, and SAXS measurements confirmed the formation of lamellar vesicles that were small in size, where the clotrimazole played a key role in their assembling and features, probably because it was well-solubilized in both vesicle compartments, i.e., the hydrating medium and phospholipid bilayer. The combination of TEM and cryo-TEM analysis allowed us to discover the native structure of dispersions in a three-dimensionally-structured network, which conferred high viscosity and stability to the system. After dilution in water media simulating the behavior in biological fluids, the network disappeared, and vesicles became akin to single particles in dispersion without undergoing the phenomena of breaking or destabilization. We can speculate that their peculiar behavior can facilitate topical delivery of the payload because the dispersion can be easily applied on the skin, avoiding the loss of the formulation after application thanks to its highly viscous structured framework. On the skin’s surface, the dispersion may interact with the components of the *stratum corneum* and then with biological fluids undergoing a dilution, which favors the penetration of single vesicles into both the *stratum corneum* and biofilm matrix. Moreover, a part of vesicles can fuse within the skin matrix, modifying its lamellar assembling and perturbing its ordered structure due to the combined action of phospholipids, glycerol, and ethanol. As confirmation of this mechanism, all hybrid vesicles, irrespective to the hydrating medium composition, were able to improve the skin accumulation of clotrimazole to a greater extent than the commercial cream Canesten^®^ used as a reference. In addition, despite the high amount of ethanol and glycerol used as a hydrating medium, the biocompatibility was very high (≈90% viability), confirming that the new vehicle used to produce hybrid vesicles did not modify the low toxicity of phospholipid vesicles. Physico-chemical and technological properties of hybrid vesicles did not display important differences among the different formulations, irrespective of the hydrating mixture used. 

In contrast, antimicrobial results confirmed the promising properties of all these new hybrid vesicles as carriers for the treatment of local *C. albicans* infections, but in particular, showed the specific activity of 59:2 and 63:10 vesicle formulations against *Candida* biofilm. These hybrid vesicles contained an intermediate amount of glycerol (≈0.61 mL) and this concentration seemed to be ideal in modulating the vesicles’ ability to interfere against biofilm formation at a low concentration. It is well known that skin candidiasis represents a “biofilm-related disease” and many studies confirmed the poor ability of azoles to counteract *C. albicans* infections structured as a biofilm. In this study, clotrimazole loaded in 59:2 and 63:10 vesicles showed a great ability to counteract *C. albicans* infections and inhibit the biofilm formation, thus underlining their promising properties, especially taking into consideration the increasing resistance of this yeast to traditional antifungal formulations and treatments [53]. 

The in vitro results were confirmed by those obtained in vivo, which underlined the higher effectiveness of the selected formulations to reduce the number of the colonies of the yeasts (CFU) in comparison with the commercial cream Canesten^®^ used as a reference. 

## 5. Conclusions

Overall, results of this study support the design of a new and promising kind of phospholipid vesicles called three-dimensionally-structured hybrid vesicles due to their original structure in dispersion, obtained by using a mixture of glycerol and ethanol with a low content of water (≤10%) as a hydrating medium. These hybrid vesicles were prepared by using an environmentally-friendly method, avoiding the use of organic solvents or dissipative methodologies. Four different formulations were obtained by changing the ratio between ethanol, glycerol, and water, which were all highly stable and able to promote the payload accumulation in the different skin strata and its antifungal activity. Results underlined that 59:2 and 63:10 hybrid vesicles contained the ideal combination of ethanol and glycerol that was capable of ensuring the highest protection against *C. albicans* biofilm formation and infection resistance. In conclusion, these hybrid vesicles may be considered as promising carriers for the treatment of topical fungal infections. 

## Figures and Tables

**Table 1:** Composition of clotrimazole loaded three-dimensionally-structured hybrid vesicles.

	Lecithin (mg/mL)	Clotrimazole (mg/mL)	Glycerol (mL)	Ethanol (mL)	Water (mL)
**59:2 vesicles**	90	10	0.59	0.39	0.02
**53:10 vesicles**	90	10	0.53	0.37	0.10
**69:2 vesicles**	90	10	0.69	0.29	0.02
**63:10 vesicles**	90	10	0.63	0.27	0.10

**Table 2:** Mean diameter, polydispersity index (PI), zeta potential, and entrapment efficiency (EE) of empty and clotrimazole-loaded three-dimensionally-structured hybrid vesicles. Mean value ± standard deviation was obtained from six independent samples.

	Mean Diameter (nm)	PI	Zeta Potential (mV)	EE (%)
Empty 59:2 vesicles	171 ± 2	0.38	−55 ± 2	-
Empty 53:10 vesicles	244 ± 13	0.43	−68 ± 3	-
Empty 69:2 vesicles	169 ± 2	0.30	−65 ± 1	-
Empty 63:10 vesicles	221 ± 3	0.38	−66 ± 3	-
Clotrimazole 59:2 vesicles	96 ± 2	0.26	−64 ± 58	96 ± 5
Clotrimazole 53:10 vesicles	186 ± 6	0.29	−59 ± 2	84 ± 5
Clotrimazole 69:2 vesicles	129 ± 7	0.28	−77 ± 1	98 ± 8
Clotrimazole 63:10 vesicles	140 ± 11	0.23	−61 ± 1	81 ± 7

**Table 3:** Fitting parameters, σ_H_ (polar head amplitude), z_H_ (position of the headgroup Gaussian of the electron density profile), and d_B_ (bilayer thickness) of empty and clotrimazole-loaded three-dimensionally-structured hybrid vesicles. Mean values ± standard deviation are reported.

	z_H_	σ_H_	d_B_
Empty liposomes	18.7 ± 0.1	3.4 ± 0.1	51.0 ± 0.6
Empty 59:2 vesicles	16.9 ± 0.2	6.5 ± 0.1	59.9 ± 0.8
Empty 53:10 vesicles	16.8 ± 0.2	6.7 ± 0.1	60.4 ± 0.8
Empty 69:2 vesicles	19.0 ± 0.1	4.7 ± 0.2	56.8 ± 1.0
Empty 63:10 vesicles	20.4 ± 0.1	4.8 ± 0.2	60.0 ± 1.0
Clotrimazole 59:2 vesicles	17.7 ± 0.2	6.3 ± 0.2	60.6 ± 1.2
Clotrimazole 53:10 vesicles	17.4 ± 0.2	6.8 ± 0.2	62.0 ± 1.2
Clotrimazole 69:2 vesicles	18.5 ± 0.2	4.5 ± 0.2	55.0 ± 1.2
Clotrimazole 63:10 vesicles	18.9 ± 0.1	4.1 ± 0.2	54.2 ± 1.0

**Table 4:** Values of MIC, MBC, and antibiofilm profile provided by clotrimazole-loaded, three-dimensionally-structured hybrid vesicles or Canesten^®^ cream. Mean values ± standard deviation are reported. Canesten^®^ values were not evaluated (NE) since it formed a turbid dispersion, which interfered with the spectrophotometric measurements.

	MIC (µg/mL)	MBC (µg/mL)	MBIC (µg/mL)
Clotrimazole 59:2 vesicles	2.5	2.5	<0.002
Clotrimazole 53:10 vesicles	>5	>5	5
Clotrimazole 69:2 vesicles	>5	>5	5
Clotrimazole 63:10 vesicles	1.25	1.25	0.004
Canesten^®^	NE	NE	NE
Empty 59:2 vesicles	>5	>5	>5
Empty 53:10 vesicles	>5	>5	>5
Empty 69:2 vesicles	>5	>5	>5
Empty 63:10 vesicles	>5	>5	>5
